# Severe Fever with Thrombocytopenia Syndrome Accompanied by Invasive Pulmonary Aspergillosis: An Autopsy Case

**DOI:** 10.3390/v13061086

**Published:** 2021-06-07

**Authors:** Kosho Iwao, Takeshi Kawaguchi, Masatoshi Kimura, Chihiro Iwao, Mao Rikitake, Ayako Aizawa, Yumi Kariya, Motohiro Matsuda, Syunichi Miyauchi, Ichiro Takajo, Takumi Kiwaki, Tsuyoshi Fukushima, Hiroaki Kataoka, Tadaki Suzuki, Akihiko Okayama, Kunihiko Umekita

**Affiliations:** 1 Department of Rheumatology Infectious Diseases, and Laboratory Medicine, Faculty of Medicine, University of Miyazaki, Miyazaki 8891692, Japan; koushou_iwao@med.miyazaki-u.ac.jp (K.I.); takeshi_kawaguchi@med.miyazaki-u.ac.jp (T.K.); masatoshi_kimura@med.miyazaki-u.ac.jp (M.K.); chihiro_kawata@med.miyazaki-u.ac.jp (C.I.); mao_komura@med.miyazaki-u.ac.jp (M.R.); ayako_kawano@med.miyazaki-u.ac.jp (A.A.); yumi_kariya@med.miyazaki-u.ac.jp (Y.K.); motohiro_matsuda@med.miyazaki-u.ac.jp (M.M.); shunichi_miyauchi@med.miyazaki-u.ac.jp (S.M.); ichiro_takajo@med.miyazaki-u.ac.jp (I.T.); okayama@med.miyazaki-u.ac.jp (A.O.); 2 Section of Oncopathology and Regenerative Biology, Faculty of Medicine, University of Miyazaki, Miyazaki 8891692, Japan; takumi_kiwaki@med.miyazaki-u.ac.jp (T.K.); fukuchan@med.miyazaki-u.ac.jp (T.F.); mejina@med.miyazaki-u.ac.jp (H.K.); 3 Department of Pathology, National Institute of Infectious Diseases, Tokyo 1620052, Japan; tksuzuki@nih.go.jp

**Keywords:** severe fever with thrombocytopenia syndrome, invasive pulmonary aspergillosis, autopsy

## Abstract

Severe fever with thrombocytopenia syndrome (SFTS) is an emerging tickborne infectious disease in China, Korea, and Japan caused by the SFTS virus (SFTSV). SFTS has a high mortality rate due to multiorgan failure. Recently, there are several reports on SFTS patients with mycosis. Here, we report a middle-aged Japanese SFTS patient with invasive pulmonary aspergillosis (IPA) revealed by an autopsy. A 61-year-old man with hypertension working in forestry was bitten by a tick and developed fever, diarrhea, and anorexia in 2 days. On day 4, consciousness disorder was appearing, and the patient was transferred to the University of Miyazaki Hospital. A blood test showed leukocytopenia, thrombocytopenia, as well as elevated levels of alanine aminotransferase, aspartate aminotransferase, lactate dehydrogenase, and creatine kinase. The SFTSV gene was detected in serum using a reverse-transcription polymerase chain reaction. On day 5, respiratory failure appeared and progressed rapidly, and on day 7, the patient died. An autopsy was performed that revealed hemophagocytosis in the bone marrow and bleeding of several organs. IPA was observed in lung specimens. SFTSV infection may be a risk factor for developing IPA. Early diagnosis and treatment of IPA may be important in patients with SFTS.

## 1. Introduction

Severe fever with thrombocytopenia syndrome virus (SFTSV) is the causative agent of severe fever with thrombocytopenia syndrome (SFTS). SFTSV is classified into the Genus *Phlebovirus*, Family *Bunyaviridae* [1], and was renamed “*Huaiyangshan banyangvirus*” *of Banyangvirus Genus of Phenuiviridae* Family by the International Committee on Taxonomy of Viruses in 2018 [2]. SFTS is characterized by the sudden onset of a fever, gastrointestinal and neural symptoms, hemorrhagic tendency, thrombocytopenia, and leukocytopenia. The laboratory findings of SFTS show elevation of alanine aminotransferase (ALT), aspartate aminotransferase (AST), lactate dehydrogenase (LDH), and creatine kinase (CK). The mortality rate in Japan is reported to be about 30% [3]. In fatal cases, disturbances of consciousness, respiratory failure, arrhythmia, kidney dysfunction, and shock are common [4]. Recently, it has been suggested that SFTS is associated with mycosis, especially invasive aspergillosis [3,4,5]. However, the pathogenesis of invasive aspergillosis in SFTS remains unclear. Herein, we present an autopsy case of SFTS complicated with invasive pulmonary aspergillosis (IPA).

## 2. Case Report

A 61-year-old man had engaged in forestry and had been repeatedly bitten by ticks on his legs throughout his life. He had taken hypertension medicine for several years. In spring, a high fever, diarrhea, appetite loss, and general fatigue occurred. Although he visited a local hospital and was started on oral antibiotics, his general condition deteriorated. Three days after the initial onset of the fever, he was admitted to a hospital. A blood test revealed leukocytopenia (0.7 × 10^9^/L), and consciousness disorder was observed. On day 4, he was transferred to the University of Miyazaki Hospital. A fresh tick-bite lesion was observed in his left calf. A left inguinal swollen lymph node, 3 cm in size, was palpable. Bleeding from his upper gums and a disturbance of consciousness were observed (Glasgow coma scale E3 V2 M4). An echocardiogram showed an ejection fraction of less than 30% and diffuse hypokinesis, suggesting myocardial dysfunction. The blood test revealed leukocytopenia, thrombocytopenia, and elevated levels of ALT, AST, LDH, and CK (Table 1). Brain natriuretic peptide (BNP) levels were elevated to 215 pg/mL, while β-D glucan was normal.

**Figure 1:**
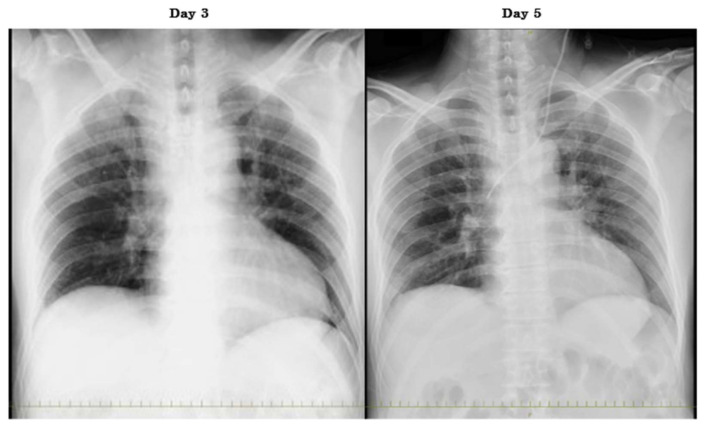
Chest radiograph. Compared to day 3, an air bronchogram appeared at the upper- and middle-lung fields on the left side on day 5.

**Figure 2:**
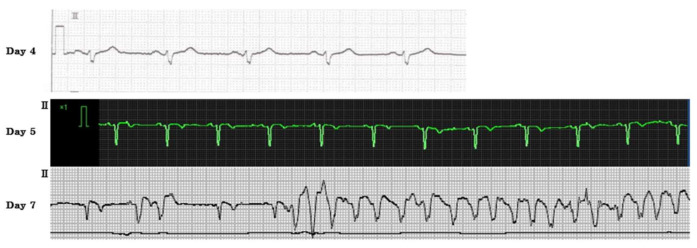
Electrocardiogram: II lead showed the right bundle branch block on day 4; monitor showed a negative T wave on day 5; sporadic ventricular tachycardia appeared on day 7.

**Figure 3:**
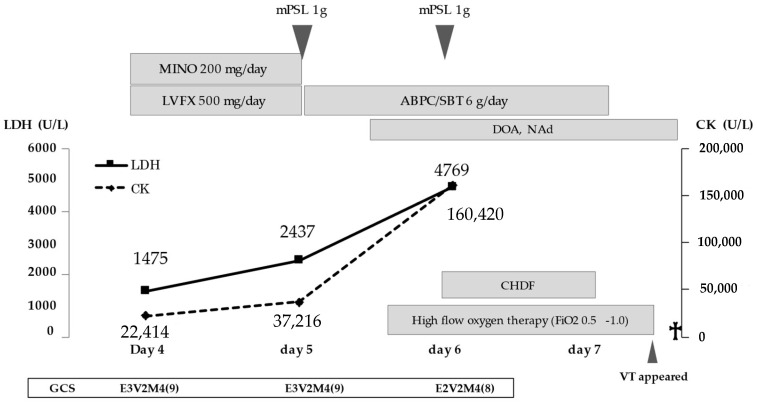
Clinical course: mPSL, methylprednisolone; MINO, minocycline; LVFX, levofloxacine; ABPC/SBT, ampicillin/sulbactam; DOA, dopamine; NAd, Noradrenaline; CK, creatine kinase; LDH, lactate dehydrogenase; CHDF, continuous hemodiafiltration; FiO2, fractional inspired oxygen concentration; GCS, Glasgow coma scale; VT, ventricular tachycardia; 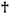: died.

Intravenous minocycline (MINO) and levofloxacin (LVFX) were started because the rickettsial infection was suspected. Five days after the initial onset of symptoms, the SFTSV gene was detected in serum by a reverse-transcription polymerase chain reaction. Therefore, this patient was diagnosed with SFTS. The viral load of SFTSV was 5.97 × 10^6^ copies/mL. We discontinued MINO and LVFX, and the patient was treated with methylprednisolone (1000 mg/day) for the virus-associated hemophagocytic syndrome. Since it was thought that he had aspiration pneumonia due to the consciousness disorder, we used ampicillin–sulbactam. There was no sputum, but respiratory failure appeared and progressed rapidly. High-flow oxygen therapy was needed. Chest radiography revealed a slight air bronchogram at the upper and middle lung field on the left side without a change of cardiothoracic ratio (Figure 1). Alveolar hemorrhage or pneumonia was suspected according to these radiological findings. The systolic blood pressure began to drop, thus a vasopressor was administered. Negative T wave first appeared on day 5 on electrocardiogram (ECG) (Figure 2). The CK-MB isozyme (CK-MB) level was 107 U/L, and this was only 0.28% in the level of CK (37,216 U/L). Due to the low proportion of CK-MB, we considered the patient to be unlikely to have myocardial damage. On day 6, because the level of CK in serum increased to 160,420 U/L and worsening renal dysfunction, continuous hemodiafiltration was started at the intensive care unit (Figure 3). On day 7, the patient’s respiratory condition did not improve despite 100% fractional inspired oxygen concentration and non-invasive positive pressure ventilation. Sporadic ventricular tachycardia (VT) also appeared (Figure 2). Although an antiarrhythmic agent was administered, the frequency of VT abnormalities was unchanged. Cardiovascular function was also deteriorating. Four hours after the appearance of VT, the patient died.

**Figure 4:**
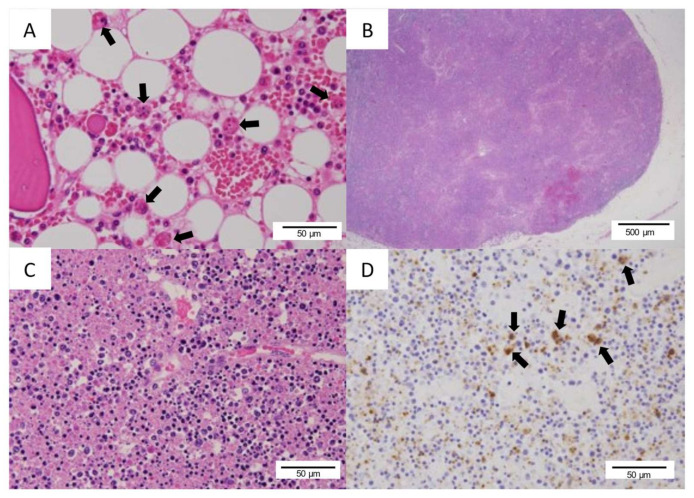
Histological findings: (**A**) arrows indicate hemophagocytosis in the bone marrow (hematoxylin–eosin stain, magnification: ×100); (**B**) the inguinal lymph node was necrotizing and lost normal architecture (hematoxylin–eosin stain, magnification: ×40); (**C**) necrotizing lymphadenitis with infiltration of histiocytes and large-sized blastoid cells (hematoxylin–eosin stain, magnification: ×100); (**D**) arrows indicate severe fever with thrombocytopenia syndrome virus–nucleoprotein (SFTSV–NP) positive cells found in the lymph node (immunohistochemistry for SFTSV–NP antigen, magnification: ×100).

**Figure 5:**
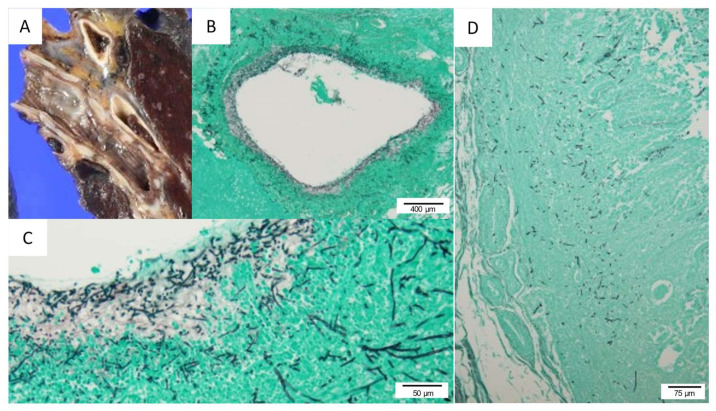
Macroscopic and histological findings: (**A**) the bronchus was covered with pseudomembranes. Erosion and necrosis were found on the bronchial wall, with sputum and necrotic material attached; (**B**) fungus invades deep into the bronchial wall (Grocott stain, magnification: ×40); (**C**) bronchial wall was destroyed by *Aspergillus* (Grocott stain, Magnification: ×100); (**D**) in the gastric mucosa, *Aspergillus* invasion was observed, and there was no ulcer lesion (Grocott stain, magnification: ×40).

An autopsy was performed six hours after his death. Gross pathological findings revealed a subcutaneous hemorrhage within prothorax regions, bilateral inguinal regions, and bilateral thigh regions. Histopathological examination revealed microscopic bleeding in multiple tissues such as skin, lungs, gastrointestinal mucosa, and thyroid and parathyroid glands. There was a marked hemophagocytosis in the bone marrow (Figure 4A). The left inguinal lymph node had lost its normal structure and presented with severe necrotizing lymphadenitis with infiltration of histiocytes and large-sized blastoid cells (Figure 4B,C). Immunohistochemistry (IHC) using an anti-SFTSV–nucleoprotein (SFTSV–NP) antibody, which was stained and analyzed by the National Institute of Infectious Diseases, revealed SFTSV–NP positive cells within the inguinal lymph node (Figure 4D), spleen, and bone marrow. Histologically, no SFTSV infection was found in the parenchymal cells of the heart and brain tissue. In gross histological findings from the myocardium, concentric left ventricular hypertrophy was observed, which was considered to be a finding of hypertensive heart disease. There were no histopathological findings suggestive of pericarditis and ischemic heart disease. The cardiac conduction system had also been preserved. Moreover, the presence of right atrial dilatation, hepatic and splenic congestion suggested right-sided heart failure. Histologically, infiltration of inflammatory cells was not apparent, and no myocarditis was observed. The trachea and bronchus were covered with pseudomembranous tissue (Figure 5A), which was accompanied by fungal infiltration.

In the left lung, upper lobe, innumerable fungus invading deep into the bronchial wall were observed (Figure 5B,C). These fungi were suspected to be *Aspergillus* according to the morphological features. Severe invasion of fungi with a destroyed bronchial wall was observed in some parts of the bronchus. Based on these findings, it was considered that IPA had developed in this patient. *Aspergillus* was also observed in the gastric mucosa (Figure 5D). According to these pathological findings, the causes of death were considered to be heart failure and respiratory failure. These findings suggested that alveolar hemorrhage, which impacted respiratory failure, might have been associated with the hemorrhagic tendency due to SFTSV infection. It is possible that both alveolar hemorrhage and IPA caused respiratory failure.

## 3. Discussion

In the present case, SFTSV infection caused severe systemic inflammation, including hemophagocytosis and the disrupted hemodynamics due to VT. The autopsy revealed that the causes of respiratory failure may be alveolar hemorrhage and IPA.

This patient showed diffuse hypokinesis via an echocardiogram, elevated BNP, abnormal ECG, and eventually, VT. These findings suggested myocardial dysfunction. Viral infection contributes to cardiomyopathy in two ways: (1) direct injury by infecting cardiomyocytes and (2) secondary immune reaction to cardiomyocytes [6]. We reviewed autopsy case reports of SFTS, which performed a virological examination (eight cases, seven reports) between 2014 and 2019 [7,8,9,10,11,12,13]. Lymph nodes were positive for SFTSV antigen in all cases tested, but the heart was positive for SFTSV antigen in three of seven cases. Lymphatic organs such as the spleen, bone marrow, and liver seem to be likely positive for SFTSV antigen. On the other hand, non-lymphatic organs are not always positive for SFTSV antigen. Regarding myocardial dysfunction, there is a report of clinically suspected myocarditis with SFTS [14] but no reports of pathologically diagnosed myocarditis. In our case, there was no infiltration of inflammatory cells and no SFTSV infection to heart parenchymal cells; therefore, it is considered that he had a myocardial dysfunction, which was not caused by viral myocarditis. Myocardial dysfunction in SFTS is likely to be the result of hypercytokinemia rather than the result of direct injury by SFTSV, and similar cases have been reported [15]. Since little is known about myocardial damage related to SFTS, it is necessary to accumulate future cases.

Both arrhythmia and respiratory failure due to IPA and alveolar hemorrhage appear to cause death for this patient. We also reviewed the prevalence of fungal infection and aspergillosis in patients who underwent autopsy [7,8,9,10,11,12,13]. Fungal infections have been demonstrated in four of nine autopsied cases, and three of four cases were complicated with aspergillosis. *Aspergillus* infiltrates into the trachea or bronchus in all three cases. This is considered to be consistent with IPA, especially *Aspergillus* tracheobronchitis (ATB). Tracheobronchitis is an uncommon manifestation of infection due to *Aspergillus* species, occurring in <7% of cases of pulmonary aspergillosis [16]. The estimated prevalence of ATB in SFTS-associated invasive aspergillosis may be as high as 60%, much greater than its prevalence in other populations [5]. Although it is difficult to make a comparative study due to the small number of cases, it is suggested that SFTS-associated IPA may tend to present with ATB.

The classical risk factors of IPA are neutropenia (<0.5 × 10^9^/L), hematological malignancy, transplantation, and prolonged treatment with corticosteroid [17]. New risk factors have been identified during the last decade, including chronic obstructive pulmonary disease, liver cirrhosis, and post-H1N1 influenza, and corticosteroid use for about seven days [17]. Our patient had two risk factors for IPA, namely, neutropenia (0.46 × 10^9^/L) and the use of corticosteroid. However, the association between the development of IPA and short-period corticosteroid use is unclear in this patient. The administration period of corticosteroid was as short as a few days to one week. In fact, a case report described the case of a patient with SFTS who developed IPA without the use of corticosteroid [5]. Therefore, it remains unclear whether corticosteroid use is one of the essential factors for the development of IPA in patients with SFTS. We also considered the relationship between neutropenia and the time course of aspergillosis infection. Among patients with autologous transplants, the median time to diagnose IPA was reported as 16 days [18]. In our case, the time of IPA development was estimated as within one week after the onset of SFTS. Therefore, there appears to be another pathogenesis of IPA development in patients with SFTS, besides neutropenia. Several reports suggested that the SFTSV has some mechanisms for escaping from a host’s immune system. First, SFTSV induced interleukin-10 production, which suppresses the immune system [19]. Second, the reduction of CD3+ and CD4+ T cells due to monocyte apoptosis by viral replication could lead to the attenuation of cellular and humoral immune responses [20]. Third, cytokine storms have been reported as a major pathophysiological mechanism that aggravates leukocytopenia and thrombocytopenia [21]. Although SFTSV infection may disturb the host immune response against *Aspergillus*, further research is necessary to clarify the pathogenesis of IPA in patients with SFTS. High viral load (>10^5^ copies/mL) has been reported to be a risk factor for death in SFTS patients [22]. The prognosis might have been poor because the present case had a high viral load of SFTSV. However, it remains unclear whether a high viral load of SFTSV is associated with the development of IPA.

We assumed that the patient had IPA and measured β-D glucan, which was negative. Only autopsy findings revealed a complication of IPA. For an early diagnosis of IPA, evaluation by computed tomography (CT) or bronchoscopy, airway specimen culture, β-D glucan, and *Aspergillus* antigen levels are essential and should be measured. However, these tests are not always positive for *Aspergillus* infection. In our case, it was difficult to evaluate IPA via chest CT and bronchoscopy because of the rapid progression of respiratory failure. In IPA, especially ATB, pseudomembrane is often formed in the bronchus. Occasionally, in cases of ATB, thickening of the trachea or bronchial wall may be apparent with high-resolution CT [23]. Tracheobronchial wall thickening on CT may help to distinguish ATB from alveolar bleeding and other bacterial pneumonia. Although chest CT may be helpful to evaluate the development of ATB in SFTS patients, it is often difficult to perform bronchoscopy and chest CT on rapidly worsening SFTS patients such as the present case.

In SFTS patients, patients with IPA have higher in-hospital mortality than those without IPA [24]. Therefore, in the management of SFTS patients, early diagnosis and treatment of IPA may provide better results. Antifungal therapy for patients with highly suspected *Aspergillus* infection is considered reasonable, but it is an important clinical issue whether to prevent IPA by antifungal agents in all SFTS cases from the early stage of onset. Non-antibiotic prevention should also be considered. Patient management via transplants in a laminar airflow (LAF) room delayed the median time to diagnosis of invasive aspergillosis by 38 days [17]. This suggests that the use of a LAF room may delay the development of aspergillosis. Still, it is challenging to use a positive pressure LAF room considering the aerosol infection of SFTS. To avoid this problem, we suggest using a high-efficiency particulate air filter in a negative pressure chamber. Since this is not an established method, it needs to be examined in more cases to determine its effectiveness.

## 4. Conclusions

We experienced an autopsy case of SFTS complicated with IPA and myocardial dysfunction. Both alveolar hemorrhage and progressive IPA might have been causes of respiratory failure. According to several autopsy reports of SFTS, it is possible that the incidence of deep fungal infection in SFTS patients is high unexpectedly. Therefore, early diagnosis and treatment of IPA may be important in patients with SFTS.

## Figures and Tables

**Table 1:** Laboratory data on the admission of our hospital.

Urinalysis		Chemistry (Reference Range)	
Protein	++++	Creatinine (0.65–1.07)	0.79 mg/dL
Sugar	-	Blood urea nitrogen (8–20)	18.1 mg/dL
Occult blood	+++	Sodium (138–145)	137 mmol/L
**Complete blood count (reference range)**		Potassium (3.4–4.8)	3.5 mmol/L
White blood cell (3.3–8.6)	0.8 × 10^9^/L	Chloride (101–108)	107 mmol/L
Neutrophil	58.6%	Total bilirubin (0.4–1.5)	0.5 mg/dL
Lymphocyte	38.7%	Aspartate aminotransferase (13–30)	564 U/L
Monocyte	2.7%	Alanine aminotransferase (10–42)	131 U/L
Eosinophil	0.0%	Lactate dehydrogenase (124–222)	1295 U/L
Basophil	0.0%	Creatine kinase (59–248)	18,419 U/L
Red blood cell (4.35–5.55)	4.92 × 10^12^/L	Total protein (6.6–8.1)	5.45 g/dL
Platelet (158–348)	45 × 10^9^/L	Albumin (4.1–5.1)	2.93 g/dL
**Coagulation system (reference range)**		C-reactive protein (0–0.14)	0.72 mg/dL
PT-INR	1.34	Brain natriuretic peptide (0–18.4)	215 pg/mL
aPTT (25–35)	140.9 sec	Serology (reference range)	
Fibrinogen (200–400)	206 mg/dL	β-d glucan (0–11)	<6.0 pg/mL
FDP (0–5)	49.4 μg/dL		

PT-INR—prothrombin time-international normalized ratio; aPTT—activated partial thromboplastin time; FDP—fibrin/fibrinogen degradation products.

## Data Availability

The data presented in this study are available on request from the corresponding author.
